# Supplementary material for: Youth engagement and social innovation in health in low-and-middle-income countries: Analysis of a global youth crowdsourcing open call
Source: PLOS Glob Public Health. 2024 Jul 18;4(7):e0003394. doi: 10.1371/journal.pgph.0003394 (PMC11257312; doi:10.1371/journal.pgph.0003394)
Supplement: S1 Fig — (DOCX) [file pgph.0003394.s002.docx]

**Supplemental Figure 1.** **Histogram showing a distribution of mean scores of entries to the Global “Go Youth” open call, 2021-2022 (n=99)**
