# Supplementary material for: Youth engagement and social innovation in health in low-and-middle-income countries: Analysis of a global youth crowdsourcing open call
Source: PLOS Glob Public Health. 2024 Jul 18;4(7):e0003394. doi: 10.1371/journal.pgph.0003394 (PMC11257312; doi:10.1371/journal.pgph.0003394)
Supplement: S1 Table — (DOCX) [file pgph.0003394.s003.docx]

**Supplemental Table 1. Submission themes for the Go Youth! Global Open Call**

| **Theme** | **Examples** |
| --- | --- |
| Innovations focused on addressing specific health topics | - Addressing the increasing incidence or spread of infectious diseases - Management of issues that are relevant to youth (including those at the interface of infectious diseases and non-communicable diseases such as obesity and mental health conditions) - Addressing the impact of epidemics/pandemics on community and/or population health |
| Innovations in changing processes, systems, and developing people-centered approaches to health | - Engaging and empowering community members (especially youth) to participate in health-enhancing activities - Developing innovations to target structures (e.g. healthcare institutions, policies, health systems, organizational practices) to improve patient and community health - De-implementation as an innovation (e.g. cessation of a programme or intervention that doesn’t work, or remains a barrier to achieving better health outcomes) |
| Innovations in campaigning or messaging for health | - Innovative campaigns or messaging (e.g., health campaigns, songs, edutainment) to enhance health-related behaviors or perceptions - Developing platforms for sharing of health knowledge and other health promotion efforts to enhance health |
