# Supplementary material for: Youth engagement and social innovation in health in low-and-middle-income countries: Analysis of a global youth crowdsourcing open call
Source: PLOS Glob Public Health. 2024 Jul 18;4(7):e0003394. doi: 10.1371/journal.pgph.0003394 (PMC11257312; doi:10.1371/journal.pgph.0003394)
Supplement: S2 Table — (DOCX) [file pgph.0003394.s004.docx]

**Supplemental Table 2. Judging Criteria for the Go Youth! Global Open Call**

| **Criteria/Scores** | **1-3** | **4-6** | **7-10** |
| --- | --- | --- | --- |
| **Clear and concise description** | The submission provides little content or description, and does not provide clear details of the rationale for the submission, the innovation itself, or its impact on health | The submission provides sufficient content to provide sufficient understanding of its rationale, the proposed innovation, and its impact on health | The submission provides a clear and concise description of the rationale for the innovation, details of the proposed innovation, and its impact on health |
| **Relevance** | The submission illustrates an innovation that may have limited impact on health or health equity | The submission illustrates an innovation that may have some impact on health or health equity | The submission illustrates an innovation that would have substantial impact on health or health equity |
| **Novelty** | The submission has limited novelty in the given setting or context and does not innovate within existing systems | The submission is somewhat novel in the given setting or context and exhibits some innovation within existing systems | The submission is novel in the given setting or context |
| **Feasibility, Scalability / Replicability and Sustainability** | There is little to no evidence to suggest that the submission is feasible or can be scaled up/replicated and sustained. | There is some evidence to suggest that the submission is feasible or can be scaled up/replicated and sustained. | There is substantial evidence to suggest that the submission is feasible or can be scaled up/replicated and sustained. |
| **Promotion of Equity and Fairness** | The submission has limited relevance to issues of equity and fairness in terms of its proposed outcomes or processes. Issues of equity are not addressed in the proposed innovation. | The submission potentially addresses issues of equity and fairness in terms of its proposed outcomes or processes. Issues of equity would potentially be addressed in the proposed innovation. | The submission address issues of equity and fairness in terms of its proposed outcomes or processes. There is strong potential to address issues of equity in the proposed innovation. |
