# Supplementary material for: Youth engagement and social innovation in health in low-and-middle-income countries: Analysis of a global youth crowdsourcing open call
Source: PLOS Glob Public Health. 2024 Jul 18;4(7):e0003394. doi: 10.1371/journal.pgph.0003394 (PMC11257312; doi:10.1371/journal.pgph.0003394)
Supplement: S3 Table — (DOCX) [file pgph.0003394.s005.docx]

**Supplemental Table 3** Overview of the themes of the entries to the Global “Go Youth” open call, 2021-2022 (n=99)

| **Category** | **Count** |
| --- | --- |
| **Innovations focused on addressing specific health topics** |  |
| Physical health and well-being | 11 |
| Mental health | 8 |
| Technological advancement | 5 |
| Social issue in general | 3 |
| Environmental issues | 1 |
| Health education | 1 |
| **Innovations in changing processes, systems, and developing people-centered approaches to health** |  |
| Physical health and well-being | 12 |
| Technological advancement | 9 |
| Social issue in general | 9 |
| Mental health | 5 |
| Health care system | 4 |
| Environmental issues | 2 |
| Health education | 1 |
| **Innovations in campaigning or messaging for health** |  |
| Health education | 22 |
| Social issue in general | 3 |
| Mental health | 2 |
| Technological advancement | 1 |
| Youth empowerment | 1 |
