# Supplementary material for: Youth engagement and social innovation in health in low-and-middle-income countries: Analysis of a global youth crowdsourcing open call
Source: PLOS Glob Public Health. 2024 Jul 18;4(7):e0003394. doi: 10.1371/journal.pgph.0003394 (PMC11257312; doi:10.1371/journal.pgph.0003394)
Supplement: S4 Table — (DOCX) [file pgph.0003394.s006.docx]

**Supplemental Table 4. Descriptive Statistics for Each Variable Stratified by Scores of Submissions in the Global “Go Youth” open call, 2021-2022 (n=99)**

|  | | **Scores of submissions** | | | | ***P* Value (Test of Independence)** |
| --- | --- | --- | --- | --- | --- | --- |
|  | **≤5**  **N(%)** | | **(5,6]**  **N(%)** | **(6,7]**  **N(%)** | **>7**  **N(%)** |  |
| **Total** | 27(27.3) | | 23(23.2) | 31(31.1) | 18(18.2) |  |
| **Age(years)** |  | |  |  |  |  |
| 19-22 | 1(11.1) | | 3(33.3) | 1(11.1) | 4(44.4) | 0.136 |
| 23-26 | 9(32.1) | | 5(17.9) | 11(39.3) | 3(10.7) |  |
| 27-30 | 7(23.3) | | 5(16.7) | 9(30) | 9(30) |  |
| >30 | 9(30) | | 9(30) | 10(33.3) | 2(6.7) |  |
| **Gender** |  | |  |  |  |  |
| Male | 18(28.1) | | 15(23.4) | 18(28.1) | 13(20.3) | 0.735 |
| Female | 8(23.5) | | 8(23.5) | 13(38.2) | 5(14.7) |  |
| **Highest degree** |  | |  |  |  |  |
| High school or the equivalent | 8(34.8) | | 6(26.1) | 7(30.4) | 2(8.7) | 0.124 |
| Bachelor's degree | 6(15.8) | | 11(29.0) | 10(26.3) | 11(29.0) |  |
| Master’s degree | 8(30.8) | | 5(19.2) | 8(30.8) | 5(19.2) |  |
| Doctoral degree | 1(14.3) | | 1(14.3) | 5(71.4) | 0(0) |  |
| Others | 3(75) | | 0(0) | 1(25) | 0(0) |  |
| **Sexuality orientation** |  | |  |  |  |  |
| Straight/Heterosexual | 22(27.9) | | 20(25.3) | 22(27.9) | 15(19) | 0.425 |
| Not Straight/Heterosexual | 4(21.1) | | 3(15.8) | 9(47.4) | 3(15.8) |  |
| **Ever participated in past social innovation in health activities** | | | | | | |
| No | 12(27.3) | | 13(30.0) | 15(34.1) | 4(9.1) | 0.166 |
| Yes | 13(25.5) | | 9(17.7) | 16(31.4) | 13(25.5) |  |
| **Prior research/implementation of innovation** | | | | | | |
| No | 12(30.8) | | 12(30.8) | 13(33.3) | 2(5.1) | 0.043 |
| Yes | 14(23.7) | | 11(18.6) | 18(30.5) | 16(27.1) |  |
