# Supplementary material for: Youth engagement and social innovation in health in low-and-middle-income countries: Analysis of a global youth crowdsourcing open call
Source: PLOS Glob Public Health. 2024 Jul 18;4(7):e0003394. doi: 10.1371/journal.pgph.0003394 (PMC11257312; doi:10.1371/journal.pgph.0003394)
Supplement: S5 Table — (DOCX) [file pgph.0003394.s007.docx]

**Supplemental Table 5. Scores for submissions based on types of youth engagement**

|  | **Number of Submissions** | **Average Judging Score** | **One-Way ANOVA p-value** |
| --- | --- | --- | --- |
| **Overall** | 99 | 5.83 | 0.137 |
| **Substantial Engagement** | 39 | 6.13 |  |
| **Moderate Engagement** | 12 | 6.22 |  |
| **Minimal Engagement** | 18 | 5.76 |  |
| **No Engagement** | 30 | 5.32 |  |
