# Supplementary material for: Youth engagement and social innovation in health in low-and-middle-income countries: Analysis of a global youth crowdsourcing open call
Source: PLOS Glob Public Health. 2024 Jul 18;4(7):e0003394. doi: 10.1371/journal.pgph.0003394 (PMC11257312; doi:10.1371/journal.pgph.0003394)
Supplement: Attachment — Submitted filename: Response to PLOS on Youth Paper.docx [file pgph.0003394.s008.docx]

**Response to PLOS on Youth Paper**

**Title: Youth Social Innovation in Health in Low-and-Middle-Income Countries: A Mixed Methods Analysis of a Global Youth Crowdsourcing Open Call**

General:

This is a very interesting topic and highlights the importance of youth driven innovations in the health sector. The paper will benefit from an improved structural sequence and consistency across all sections as well as more appropriate interpretation of the meaning and significance of findings. The paper is also a mix of a descriptive study and a program-based description of an activity. Readers will benefit from a better delineation of the different aspects; the study and the intervention (capacity development) including a clear description of whether the impact of the program was assessed as part of the study. Specific details are outlined below by section.

1. Abstract:

- There is some inconsistency in the flow of the abstract. While the introduction stated that the study seeks to examine quality and extent of youth engagement in social innovations, the methods and results sections of the abstract were silent on how quality was assessed and what was found. The conclusion then states that youths developed and implemented high quality social innovations and concluded on the feasibility of social innovations from LMICs which could not have been derived from the results as stated above. All sections should be revised for flow and consistency.

2. Methods:

- Please provide a more detailed explanation of the mixed methods. What was qualitative and what was quantitative?
- How was the call for application targeted at youths? It will be important to state that youth friendly media were used to attract the youth population if so. Perhaps also include scientific reference that shows that the youth could be reached through these media forms.
- Was there a criterion for selecting the assessment committee?
- Provide a better description of how quality of social innovation was assessed. The whole document will benefit better from a good description of how the quality of the social innovation was assessed; including clear methods (aside from the table in the annexures) including if it is derived from an existing framework, tool, or method, or if it was developed for the purpose of the assessment. In which case, kindly provide justification or scientific evidence for the criteria used to measure quality.

3. Results

- Provide more clarity on the stage where the capacity development was done, did the capacity development affect the study? Was there a pre and post capacity development submission? Or was the assessment based on the initial submissions before the capacity development? If so, please kindly explain the reasons for inclusion of the capacity assessment in this article. Did the capacity development increase youth engagement or quality and in what way or by what unit points or percentage? It may be better to describe the results in two parts: (i) the findings from analysing the submissions using a quality and youth engagement criteria and (i), a description of the intervention program that happened after the assessment, including the sequence of events.
- Please kindly provide a better description of the quality of the submissions of the social innovations, a big part of the results was focused on youth engagement with inadequate information and data on the quality part of the findings.
- Did the authors try to explore possible reasons for lower female response? Was this similar in other studies?

4. Conclusion

- The study concluded that there was exceptional youth social innovation, this could not be deduced from the body of the document (refer to earlier comment on describing the quality methodology and results better). What is the data to back up the exceptional? How is exceptional defined?
- There were some descriptions of the approach in the conclusion page “ie scaffolding approach”, this would fit better in the methodology section.
