# Supplementary material for: Youth engagement and social innovation in health in low-and-middle-income countries: Analysis of a global youth crowdsourcing open call
Source: PLOS Glob Public Health. 2024 Jul 18;4(7):e0003394. doi: 10.1371/journal.pgph.0003394 (PMC11257312; doi:10.1371/journal.pgph.0003394)
Supplement: Attachment — Submitted filename: Response to Reviewers_PGPH-D-23-02030.docx [file pgph.0003394.s009.docx]

**Response to Reviewers**

**Manuscript Number:** PGPH-D-23-02030

**Manuscript Title:** Youth Engagement and Social Innovation in Health in Low-and-Middle-Income Countries: Analysis of a Global Youth Crowdsourcing Open Call

**Dear Dr. Eboreime,**

Thank you very much for the opportunity to revise and resubmit our manuscript for consideration at PLOS Global Public Health. We thank the reviewers for the constructive and thoughtful comments. We have considered the comments and revised the manuscript in response to them.

We have also amended the title slightly to better reflect the details of our manuscript: “Youth Engagement and Social Innovation in Health in Low-and-Middle-Income Countries: Analysis of a Global Youth Crowdsourcing Open Call”

Please find in this note a brief explanation of the revisions made in response to the reviewer’s comments.

**Comments from Editorial Team**

1. Please include a complete copy of PLOS’ questionnaire on inclusivity in global research in your revised manuscript. Our policy for research in this area aims to improve transparency in the reporting of research performed outside of researchers’ own country or community. The policy applies to researchers who have travelled to a different country to conduct research, research with Indigenous populations or their lands, and research on cultural artefacts. The questionnaire can also be requested at the journal’s discretion for any other submissions, even if these conditions are not met. Please find more information on the policy and a link to download a blank copy of the questionnaire here: https://ddec1-0-en-ctp.trendmicro.com:443/wis/clicktime/v1/query?url=https%3a%2f%2fjournals.plos.org%2fglobalpublichealth%2fs%2fbest%2dpractices%2din%2dresearch%2dreporting&umid=1ca1fdd2-e818-43b6-a7a0-b76b7bcb8903&auth=8d3ccd473d52f326e51c0f75cb32c9541898e5d5-947629db1de9a938941415ff90e05bcdfd725590. Please upload a completed version of your questionnaire as Supporting Information when you resubmit your manuscript.

**Response:** We thank the editors for the comment. We have now included the questionnaire as supporting information in our resubmission.

2. Please send a completed 'Competing Interests' statement, including any COIs declared by your co-authors. If you have no competing interests to declare, please state "The authors have declared that no competing interests exist". Otherwise please declare all competing interests beginning with the statement "I have read the journal's policy and the authors of this manuscript have the following competing interests:"

**Response:** We thank the editor for the opportunity to clarify this. We have now included a competing interests statement in our manuscript.

3. Please amend your detailed Financial Disclosure statement. This is published with the article. It must therefore be completed in full sentences and contain the exact wording you wish to be published.

a. State the initials, alongside each funding source, of each author to receive each grant.

b. If any authors received a salary from any of your funders, please state which authors and which funders.

If you did not receive any funding for this study, please simply state: “The authors received no specific funding for this work.

**Response:** We thank the editor for the opportunity to clarify this. We have now clarified the funding source alongside the initials of co-authors who received funding for this work.

4. In the online submission form, you indicated that "Data is available from the corresponding author (RT) upon reasonable request". All PLOS journals now require all data underlying the findings described in their manuscript to be freely available to other researchers, either 1. In a public repository, 2. Within the manuscript itself, or 3. Uploaded as supplementary information.

This policy applies to all data except where public deposition would breach compliance with the protocol approved by your research ethics board. If your data cannot be made publicly available for ethical or legal reasons (e.g., public availability would compromise patient privacy), please explain your reasons by return email and your exemption request will be escalated to the editor for approval. Your exemption request will be handled independently and will not hold up the peer review process, but will need to be resolved should your manuscript be accepted for publication. One of the Editorial team will then be in touch if there are any issues.

**Response:** We thank the editors for clarifying this requirement. Provision of individual level responses would breach the consent terms and approved ethics protocol. We will explain these reasons in our return email accordingly.

**Comments from Reviewer 1**

1. In the introduction section, regarding “There is growing evidence that social innovation in health approaches can improve health outcomes2 3 and address the upstream determinants of health in low and middle income countries”, please elaborate on how this has been done?

**Response:** We thank the reviewer for the opportunity to clarify this. We have now included brief descriptions of these examples to elaborate on this point:

*“Examples include the use of crowdsourcing to develop effective community-driven, bottom-up solutions, or the use of crowdfunding and public engagement platforms in LMIC settings to fund community-driven health research.”*

2. You mentioned three themes in this youth global open call, and I'm curious how you ensured the rigor of the judging? Did each judge randomly review all three categories? How did you ensure that all three categories were evaluated in the fairest way possible? How do you ensure that the judges are consistent in their understanding of the rating scale?

**Response:** We thank the reviewer for the opportunity to clarify the judging process. We have now clarified that the categories or topics were guidelines for what we would accept as content for the submission; however participants’ submissions were not judged according to categories. We have now also revised the text to provide more detail on the judging process. We believe that the random assignment, declaration of conflicts of interest, purposeful selection of diverse, expert judges, and ensuring multiple scores (and additional ones if standard deviation exceed two), has helped to strengthen fairness in the judging process:

*“A judging panel was convened to assess each submission and was comprised of health professionals, researchers, youth community leaders and social innovators. Judges were selected based on their expertise in working with youth in the community, experience with implementing social innovations in health, as well as health systems researchers and professionals who have experience in research on social innovations in health. Seventeen independent judges used pre-defined judging criteria which were developed by the steering committee to evaluate submissions, including (i) clarity and conciseness, (ii) relevance, (iii) novelty, (iv) feasibility, scalability/replicability and sustainability, and (v) promotion of equity and fairness (Supplemental Table 2). Each judge was briefed on the judging criteria, and confirmed that they had understood the criteria and scoring protocol prior to the judging process. All submissions were randomly assigned to judges for scoring. Non-English submissions were translated to English for judging. Multimedia submissions were transcribed. Judges were asked to provide a single score of 1 to 10 and were asked to recuse themselves from submissions where a conflict of interest was identified. Each submission received at least three scores, and additional judges were assigned to submissions where scores exceeded a standard deviation of two. A total of eleven semi-finalists were identified following the judging process (three participants were tied in ninth place, and therefore there was a total of 11 submissions selected for the semi-finals).”*

3. It seems like most of the discussion centered around this contest would increase youth engagement in health topics, but what are its implications for LMIC settings? In other words, what is the significance of this open call, the submissions and the findings? It should not merely complement the existing crowdsourcing contests.

**Response:** We thank the reviewer for the comment and for the opportunity to discuss how the contest has implications for LMIC settings. We have now included additional reflections in our final section on how the crowdsourcing open call can help identify not just youth-led, but ground-up innovations that have the potential to transform healthcare delivery at the local level:

*“This study also provides further evidence for the feasibility of social innovations to spur health in LMICs, where top-down funding and resourcing for the strengthening of health systems may be suboptimal. The crowdsourcing open call has identified social innovations that are bottom-up and driven by youth in LMICs and have preliminary data supporting the effectiveness of their innovations. Such efforts can be further cultivated to drive innovation and transform healthcare delivery systems at the local level.”*

**Comments from Reviewer 2**

1. This is a very interesting topic and highlights the importance of youth driven innovations in the health sector. The paper will benefit from an improved structural sequence and consistency across all sections as well as more appropriate interpretation of the meaning and significance of findings. Readers will benefit from a better delineation of the different aspects; the study and the intervention (capacity development) including a clear description of whether the impact of the program was assessed as part of the study. Specific details are outlined in the attached document.

**Response:** We would like to thank the reviewer for the comments. Please find our response to the specific comments in the following section.

2. Abstract:

There is some inconsistency in the flow of the abstract. While the introduction stated that the study seeks to examine quality and extent of youth engagement in social innovations, the methods and results sections of the abstract were silent on how quality was assessed and what was found. The conclusion then states that youths developed and implemented high quality social innovations and concluded on the feasibility of social innovations from LMICs which could not have been derived from the results as stated above. All sections should be revised for flow and consistency.

**Response:** We would like to thank the reviewer for the constructive comments. We agree that revising the flow of the abstract, and ensuring its alignment with the stated methods and structure of the subsequent sections, would improve the flow and consistency of the paper. We have made the following specific edits in an attempt to achieve this:

Abstract: We have now amended the aim or objective of this study to align the aims with out methods that included a descriptive analysis of quantitative data, as well as a qualitative analysis:

*“The purpose of this mixed methods study is to describe the findings of a global youth (18-35 years old) crowdsourcing open call on social innovations, and to identify the levels of engagement in such innovations.”*

Conclusion: While our study did not evaluate the impact or effectiveness of these social innovations, this open call served to recognize innovations that already had demonstrated impact or effectiveness through the availability of preliminary data. To ensure that the language of our manuscript better reflects this, we have made some minor revisions in our conclusion section.

*LMIC youth developed and implemented social innovations that had evidence of impact or effectiveness in their communities, illustrating how social innovation approaches may be feasible in LMICs. Capacity-building activities are also feasible and can better prepare participants for submissions in crowdsourcing open calls. More efforts should be made to identify and empower youth in these settings to spark change.*

Additionally, we have now restructured our conclusion section to firstly highlight how the open call was able to (i) identify pre-existing youth-led social innovations in LMICs, (ii) show varying depths of youth engagement, as well as (iii) how capacity building can be incorporated as part of the open call process [adding to the literature on crowdsourcing open calls in LMICs].

3. Methods:

Please provide a more detailed explanation of the mixed methods. What was qualitative and what was quantitative?

**Response:** We thank the reviewer for this comment. Upon re-reading the manuscript, we realize that the use of ‘mixed-methods’ may not accurately reflect the nature of our analysis. While we adopted quantitative (to describe submissions) and qualitative approaches (to identify levels of youth engagement), we did not make any effort to ‘mix’ them or integrate them, as a mixed-methods study would typically do. As such, we have now removed mentions of the term ‘mixed methods’ and have simply described the quantitative aspects and the qualitative aspects separately for better clarity.

4. How was the call for application targeted at youths? It will be important to state that youth friendly media were used to attract the youth population if so. Perhaps also include scientific reference that shows that the youth could be reached through these media forms.

**Response:** We thank the reviewer for the comment and for the opportunity to clarify this. We have now included details on how we disseminated this global open call through our partner organizations:

*“Our team partnered with 20 organizations (Supplemental Figure 1) to promote the open call. These organizations typically serve youth in their own contexts and have experience tailoring promotional material to better resonate with their own youth members.”*

5. Was there a criterion for selecting the assessment committee?

**Response:** We would like to thank the reviewer for the opportunity to clarify this. We have now added more detail to how our judges were selected:

*“A judging panel was convened to assess each submission and was comprised of health professionals, researchers, youth community leaders and social innovators. Judges were selected based on their expertise in working with youth in the community, experience with implementing social innovations in health, as well as health systems researchers and professionals who have experience in research on social innovations in health.”*

6. Provide a better description of how quality of social innovation was assessed. The whole document will benefit better from a good description of how the quality of the social innovation was assessed; including clear methods (aside from the table in the annexures) including if it is derived from an existing framework, tool, or method, or if it was developed for the purpose of the assessment. In which case, kindly provide justification or scientific evidence for the criteria used to measure quality.

**Response:** We would like to thank the reviewer for the comment and opportunity to clarify this. We apologize for the earlier use of the phrase ‘high-quality’, as this may imply that our manuscript adopted methods that could evaluate the quality of specific social innovations that were submitted to our open call. Our intention was to highlight that the submissions included evidence of impact or effectiveness in the local communities, and the judging process took that into consideration as part of the judging rubric. As such, we have now avoided the use of the term or phrase ‘high-quality’.

7. Results

Provide more clarity on the stage where the capacity development was done, did the capacity development affect the study? Was there a pre and post capacity development submission? Or was the assessment based on the initial submissions before the capacity development? If so, please kindly explain the reasons for inclusion of the capacity assessment in this article. Did the capacity development increase youth engagement or quality and in what way or by what unit points or percentage? It may be better to describe the results in two parts: (i) the findings from analysing the submissions using a quality and youth engagement criteria and (i), a description of the intervention program that happened after the assessment, including the sequence of events.

**Response:** We thank the reviewer for this important comment. We agree that the inclusion of the capacity-building component distracts the reader from the main contribution of this paper, which was to illustrate the breadth of our submissions as well as the depth of youth engagement highlighted in these submissions. We have still retained a description of the capacity building components in our methods section:

*“As shown in Figure 1, three additional capacity building activities were conducted to scaffold our approach to the entire open call process. This is a novel component of our crowdsourcing open call that builds on the existing conceptual framework and stages of crowdsourcing from the WHO practical guide for crowdsourcing in health.15 We adopted a scaffolding approach, where multiple stages of learning and key milestones are incorporated to provide the necessary assistance to enable participants and learners to accomplish tasks and develop an understanding of the learning material.”*

But have now revised the manuscript to omit the potential impact of such capacity-building activities, given that there was no evaluation component suggesting if this was effective or not. Given that this is a novel aspect of our crowdsourcing open call relative to the WHO guide, we have revised our discussion paragraph to discuss how this may warrant further study:

*“Finally, our open call included more capacity building activities compared to other open calls. This was especially important because about half of the youth reported participating in social innovation in health activities prior to the open call. In the context of the crowdsourcing open call, the first participatory webinar aimed to clarify concepts around the judging criteria and provide participants a space to clarify doubts with their submissions. The practice pitch then provided semi-finalists the opportunity to prepare for their final pitch in terms of the format and process, as well as get structured feedback from mentors and other peers. The mini bootcamp focused on crowdfunding components that allowed participants to consider how their respective pitches could incorporate elements of crowdfunding. Finally, seed funding was given to the top three submissions to spur further research and scaling up. Further research on how such capacity-building activities better improve outcomes for crowdsourcing open calls and build capacity for youth in LMIC settings is warranted.”*

8. Please kindly provide a better description of the quality of the submissions of the social innovations, a big part of the results was focused on youth engagement with inadequate information and data on the quality part of the findings.

**Response:** We would like to thank the reviewer for the comment and opportunity to clarify this. As mentioned earlier, we apologize for the earlier use of the phrase ‘high-quality’, as this may imply that our manuscript adopted methods that could evaluate the quality of specific social innovations that were submitted to our open call. Our intention was to highlight that the submissions included evidence of impact or effectiveness in the local communities, and the judging process took that into consideration as part of the judging rubric. As such, we have now avoided the use of the term or phrase ‘high-quality’.

9. Did the authors try to explore possible reasons for lower female response? Was this similar in other studies?

**Response:** We thank the reviewer for this comment and for the chance to reflect and respond to this. While participants could submit entries in teams of up to four participants only the submitting participants’ demographics were recorded. As such, it might have been the case that the overall demographic of participants may differ from those of the submitting participants. We have now included this reflection in our limitations section:

“*Third, we note that there were certain demographic imbalances in those who submitted to our open call, including greater representation from those who were of the male gender, and those with at least a Bachelor degree and above. These reflected demographics of the submitting participant, but we did not collect information from other team members. Future calls should consider collecting more detailed information from participants to better elucidate potential inequities in the submissions process.*”

10. Conclusion

The study concluded that there was exceptional youth social innovation, this could not be deduced from the body of the document (refer to earlier comment on describing the quality methodology and results better). What is the data to back up the exceptional? How is exceptional defined?

**Response:** We thank the reviewer for the comment and the opportunity to clarify this. We note the issues around describing the quality of submissions when no evaluative aspect was included in our study to do so. We have now removed the term ‘exceptional’ from this paragraph.

11. There were some descriptions of the approach in the conclusion page “ie scaffolding approach”, this would fit better in the methodology section.

**Response:** We thank the reviewer for the comment – we have now shifted this portion to our methods section instead.

**Comments from Reviewer 3**

1. I have several suggestions for improvement:

In paragraph 3 of the introduction: The author begins each sentence with "Crowdsourcing". Please consider rewriting this paragraph to enhance readibility

**Response:** We thank the reviewer for the comment and for the chance to improve the flow and clarity of our introduction. We have now revised this paragraph accordingly:

*“Crowdsourcing has non-experts and experts generate solutions to a problem, then share selected solutions with the broader public. This approach has been used to improve health outcomes and tap collective wisdom, engage communities in diverse settings, and identify high-quality innovations. Crowdsourcing open calls have been used to develop research mentorship strategies, inform novel health interventions, and inform health policy.”*

2. Regarding the purpose of the study, one aspect is "to describe the extent of youth engagement in the innovations." In this context, could you clarify who the youth are? Are they the members of the implementing team or the youth affected by the interventions?

**Response:** We thank the reviewer for the comment. We have now revised our methods section under ‘qualitative data generation and analysis’ to better describe this approach:

*“We conducted a qualitative data analysis to identify and understand the depth of youth engagement in the open call submissions. While all of the description of innovations were submitted by youth, this analysis was especially focused on how these youth-designed innovations engaged other youth in their communities. To do so, we analyzed two sources of data within the submissions; first, we analyzed responses to a question that asked participants about how youth were engaged in the submission, and second, we analyzed the innovation descriptions themselves.”*

3. Results/Discussion

The majority of participants (70%) were from Africa. How do the characteristics of these participants influence the study results? Are there any differences in the type of engagement or innovations observed among non-African participants?

**Response:** We would like to thank the reviewer for the comment and for the chance to clarify this. We have done a calculation and found that the median scores of submissions do not significantly differ between African (6.1) versus non-African (5.9) submissions. As our study was focused largely on the depth of youth engagement, we have now included additional discussion on how future studies could conduct comparative analyses:

*“In conclusion, future research could be done in multiple areas. First, research should focus on how capacity building activities in crowdsourcing can be evaluated to support such social innovation activities and build capacity for LMIC social innovators; second, further work could be done to compare how youth engagement or social innovations may differ from one setting or context to others; third, more work can be done in the monitoring and evaluation of such innovations identified by crowdsourcing open calls and to support the sustainable implementation of such innovations in the long run.”*

4. The article mentions that this open call included more capacity building compared to other open calls. Could you explain the rationale behind comparing this call with others? How can you justify that it has a greater capacity-building component?

**Response:** We thank the reviewer for the comment and for the opportunity to clarify this. We have now explained this in our methods section:

*As shown in Figure 1, three additional capacity building activities were conducted to scaffold our approach to the entire open call process. This is a novel component of our crowdsourcing open call that builds on the existing conceptual framework and stages of crowdsourcing from the WHO practical guide for crowdsourcing in health.15 We adopted a scaffolding approach, where multiple stages of learning and key milestones are incorporated to provide the necessary assistance to enable participants and learners to accomplish tasks and develop an understanding of the learning material.*

We now describe the potential of further research for such capacity-building activities in our conclusion section:

*“Finally, our open call included more capacity building activities compared to other open calls. This was especially important because about half of the youth reported participating in social innovation in health activities prior to the open call. In the context of the crowdsourcing open call, the first participatory webinar aimed to clarify concepts around the judging criteria and provide participants a space to clarify doubts with their submissions. The practice pitch then provided semi-finalists the opportunity to prepare for their final pitch in terms of the format and process, as well as get structured feedback from mentors and other peers. The mini bootcamp focused on crowdfunding components that allowed participants to consider how their respective pitches could incorporate elements of crowdfunding. Finally, seed funding was given to the top three submissions to spur further research and scaling up. Further research on how such capacity-building activities better improve outcomes for crowdsourcing open calls and build capacity for youth in LMIC settings is warranted.”*

5. How do the characteristics of the participants affect the types of innovation observed?

**Response:** We thank the reviewer for the comment – we have responded to this comment in a similar one above.
